# Repair Process Impairment by *Pseudomonas aeruginosa* in Epithelial Tissues: Major Features and Potential Therapeutic Avenues

**DOI:** 10.3389/fcimb.2019.00182

**Published:** 2019-05-31

**Authors:** Manon Ruffin, Emmanuelle Brochiero

**Affiliations:** ^1^Centre de Recherche du Centre Hospitalier de l'Université de Montréal (CRCHUM), Montréal, QC, Canada; ^2^Département de Médecine, Université de Montréal, Montréal, QC, Canada; ^3^INSERM, Centre de Recherche Saint-Antoine, CRSA, Sorbonne Université, Paris, France

**Keywords:** *Pseudomonas aeruginosa* infections, epithelial repair, wound repair, skin, burn, lung, airway, cornea

## Abstract

Epithelial tissues protecting organs from the environment are the first-line of defense against pathogens. Therefore, efficient repair mechanisms after injury are crucial to maintain epithelial integrity. However, these healing processes can be insufficient to restore epithelial integrity, notably in infectious conditions. *Pseudomonas aeruginosa* infections in cutaneous, corneal, and respiratory tract epithelia are of particular concern because they are the leading causes of hospitalizations, disabilities, and deaths worldwide. *Pseudomonas aeruginosa* has been shown to alter repair processes, leading to chronic wounds and infections. Because of the current increase in the incidence of multi-drug resistant isolates of *P. aeruginosa*, complementary approaches to decrease the negative impact of these bacteria on epithelia are urgently needed. Here, we review the recent advances in the understanding of the impact of *P. aeruginosa* infections on the integrity and repair mechanisms of alveolar, airway, cutaneous and corneal epithelia. Potential therapeutic avenues aimed at counteracting this deleterious impact of infection are also discussed.

## Introduction

*Pseudomonas aeruginosa* is an opportunist pathogen that preferably infects immunocompromised or hospitalized patients and causes more or less severe, local to systemic, infections that can lead to death. This pathogen has minimal nutritional needs and can adapt to a wide variety of environmental conditions. Treatment of *P. aeruginosa* infections with antibiotics became often difficult because of the high potential of this pathogen to develop resistance, thus adding to its pathogenicity. Multi-drug resistant strains of *P. aeruginosa* have been classified by several healthcare organizations as serious threat to public health. Considering its high prevalence with associated high mortality rates and limited treatment options, this pathogen has been identified as a critical research priority for the development of novel therapies (Tacconelli et al., 2017; World Health Organization, 2017).

*Pseudomonas aeruginosa* infects various organs such as skin, ear, eye, heart, blood, soft tissue, or bone and joints and respiratory, urinary, gastrointestinal and central nervous systems. However, epithelial barriers of the skin, eyes, and respiratory tract, which are in direct contact with the external environment, have increased probability of infection. Thus, cutaneous, corneal, airway and alveolar infections are of particular concern considering their prevalence. *P. aeruginosa* infections of the cutaneous epithelium occur mostly after injuries. Thus, burn victims and patients with chronic cutaneous wounds, a common complication of diabetes, are particularly at risk for developing *P. aeruginosa* infections (Weinstein and Mayhall, 2003; Mihai et al., 2014). Sight-threatening invasive *P. aeruginosa* corneal epithelial infections can occur in patients using contact lenses or after corneal trauma (Green et al., 2008). Finally, acute pneumonia and chronic airway epithelial infections are frequent in mechanically ventilated or immunocompromised patients and in patients with cystic fibrosis (CF) or chronic pulmonary obstructive disease (COPD) (Parker et al., 2008; Murphy, 2009; Fujitani et al., 2011; Heltshe et al., 2018).

*Pseudomonas aeruginosa* has an opportunistic behavior, exploiting breaches in host defenses to initiate and establish infections. Furthermore, there is a growing body of evidence that *P. aeruginosa* is not only responsible for epithelial damage (section Evidence for Epithelial Integrity Alterations by *Pseudomonas aeruginosa*) but also impairs epithelial repair mechanisms after injury (section Evidence for Epithelial Repair Impairment by *Pseudomonas aeruginosa*). Indeed, while uninfected epithelia are able to set up an organized process of complex mechanisms to restore epithelial integrity and function after injury, the presence of *P. aeruginosa* restrains the ability of epithelial tissues to repair adequately, leading to persistent infections and altered organ function. As detailed below, evidence for the negative impact of *P. aeruginosa* on epithelial integrity and repair processes have been highlighted by several groups worldwide, studying various pathologies, species, organs and epithelia.

Here, we review the recent advances in our understanding of the impact of *P. aeruginosa* infections, including the bacterial factors and molecular mechanisms responsible, on epithelial integrity and repair mechanisms with a particular emphasis on the epithelia of the respiratory tract (alveoli, airways), skin and cornea. What can we learn from the similarities and differences, between alveolar, airway, cutaneous and corneal epithelial responses during repair, in the presence of *P. aeruginosa* infection? Then, we discuss the progress made in pulmonary, dermatology and ocular research in the identification of therapeutic strategies aimed at counteracting the deleterious effects of *P. aeruginosa* on the repair of airway, cutaneous and corneal epithelia.

## *Pseudomonas aeruginosa* Infections in Cutaneous, Corneal, Airway, and Alveolar Epithelia

### Common Structural Features and Differences Between Healthy Cutaneous, Corneal, and Respiratory Epithelia

The common structural and functional characteristics of epithelia allow them to act as a barrier and protection against environmental particles and pathogens. However, cutaneous, corneal, airway and alveolar epithelia also have specific features in healthy conditions, as described below, adapted to their environment and function, which also confer them specific sensitivity and ability to respond to external aggressions.

**Figure 1:**
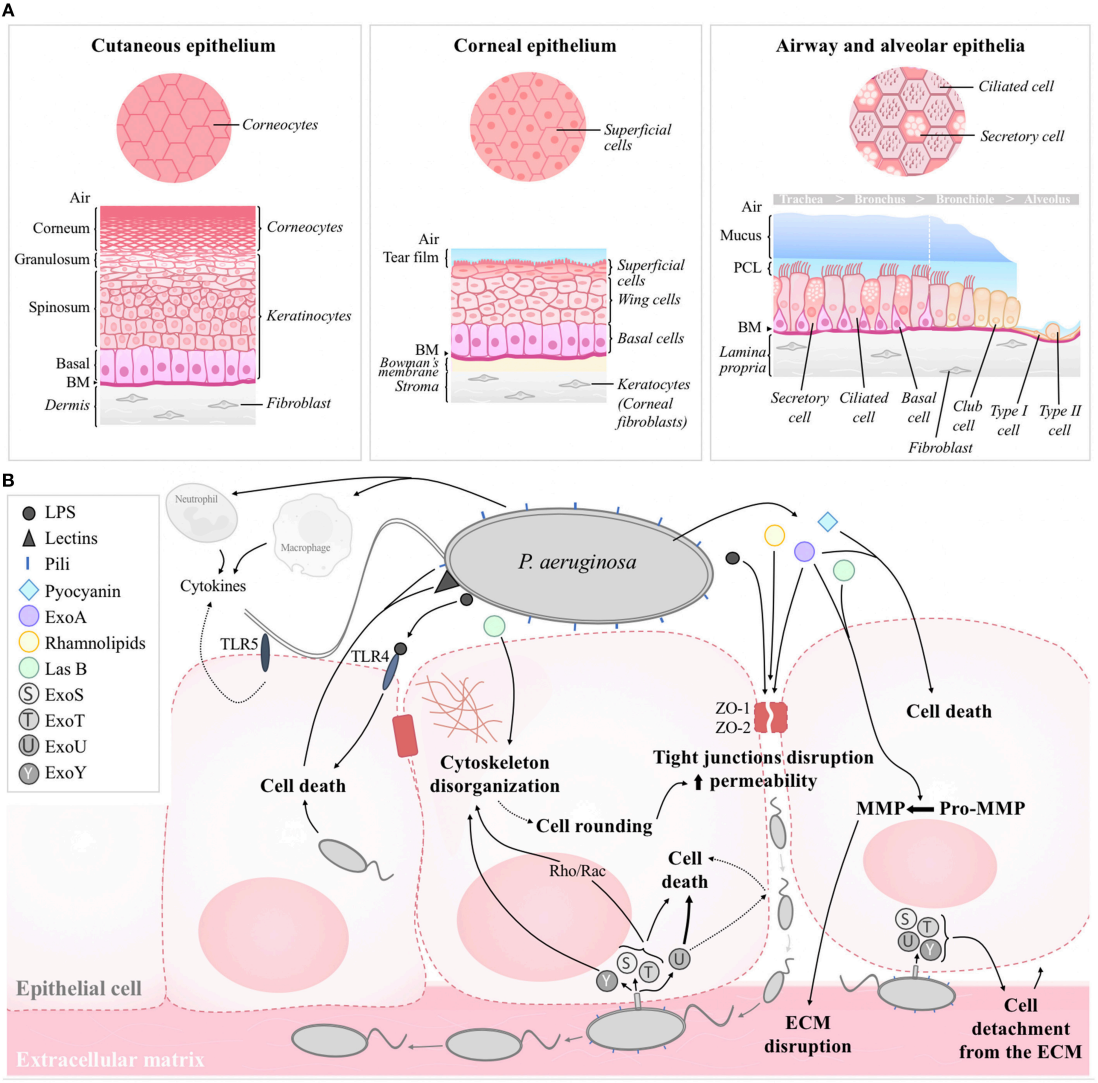
**(A)** Structural organization of the human cutaneous, corneal, airway, and alveolar epithelia in healthy conditions. **(B)** Schematic model of the effects of *P. aeruginosa* on epithelial integrity. Structural constituents (pili, LPS, lectins) and secreted factors (e.g., pyocyanin, ExoA, LasB, rhamnolipids) contributes to cell death, cytoskeleton disorganization and/or tight junction disruption. Paracellular permeability then favors bacterial transmigration and infiltration toward the basolateral compartment; ECM disruption by MMP and cell detachment to the ECM then favor bacterial propagation. T3SS toxins (e.g., ExoS, ExoT, ExoU, ExoY) injected through the basolateral membrane contribute to cell death and altered epithelial integrity. Pili and flagellum are also involved in bacterial adherence to the host cell. Cytokines, released by epithelial cells, macrophages and infiltrated neutrophils also contribute to epithelial damage. BM, basement membrane; ASL, airway surface liquid; ECM, extracellular matrix.

Structurally, cutaneous and corneal epithelia are both made of multiple cell layers while the epithelium lining the conducting airways, from the nasal fossa to the bronchi, is qualified as pseudostratified (Table 1 and Figure 1A). The simple squamous structure of the respiratory epithelium, lining alveoli, facilitates gas exchange but makes this thin barrier highly sensitive to aggressions. On the contrary, the cutaneous epithelium, exposed continuously to physical, chemical, and biological aggressions from the environment, is the thickest of these four epithelia (Table 1). For their part, corneal and airway epithelia are covered by a protective liquid phase. While the corneal epithelium is overhung by an aqueous liquid called tear film, the airway epithelium is covered by the airway surface liquid (ASL) comprising of periciliary liquid layer (PCL) and mucus layer (containing mucins) (Figure 1A).

**Table 1:** Compared characteristics and experimental *in vitro* and *in vivo* models of cutaneous, corneal, airway, and alveolar epithelia.

	**Skin**	**Eye**	**Respiratory system**	
	**Cutaneous epithelium**	**Corneal epithelium**	**Airway epithelium**	**Alveolar epithelium**
**Epithelium type**	**Stratified squamous keratinized**	**Stratified squamous non-keratinized**	**Pseudostratified columnar non-keratinized**	**Squamous monolayer**
**Thickness**	≈ 60–100 μm	≈ 45–60 μm	≈ 20–40 μm	<1 μm
**Experimental models**	Human cutaneous tissue; *in vivo* animal (wt/diabetic/CF mouse, rat, porcine) models of acute or chronic (biofilm) infection with mono- or mixed-species of dorsal or latero-abdominal (full- or partial-thickness) wound/burn skin; dermal full-thickness, rabbit ear excisional wound model of acute and chronic biofilm infection; primary human keratinocytes and dermal fibroblast; human keratinocyte cell lines (HaCaT…)	*In vivo* null-infection models; contact lens-wearing *in vivo* models in rabbits, rats, guinea pigs and mice; *in vivo* scarification and epithelial healing models of *P. aeruginosa* keratitis; e*x vivo* corneal models of *P. aeruginosa* epithelial colonization and traversal; rat/rabbit/mouse corneal tissues; human corneal tissues; exfoliated human corneal epithelial cells; primary cultured corneal epithelial cells; human corneal epithelial cell lines	*In vivo* animal (wt or CF mouse, porcine) models of acute or chronic infection (intra-tracheal or intra-nasal instillation); *ex vivo* infection of porcine lung tissues; *in situ* infection of tracheal tissues; primary human bronchial, tracheal, nasal and alveolar epithelial cell cultures (monolayers/differentiated); human lung epithelial cell lines (CFBE; A549, H292, H1299, H1975, Calu3, UNCN1T, and UNCN2T, 16HBE)	

Cell type compositions of the cutaneous, corneal, airway and alveolar epithelia are represented on the schematic models in Figure 1A. The most apical layer of the cutaneous epithelium, named stratum corneum, is composed of dead cells, called corneocytes. These cells, without nuclei and cytoplasm but containing keratin fibers, confer protection against the environmental aggressions and prevent dehydration of the skin tissue. Under the stratum corneum, live keratinocytes, the main cell type of the cutaneous epithelium, form several layers (Figure 1A). The multilayered corneal epithelium is composed of three different cell types: superficial cells, wing cells and basal cells, from the apical to the basal side (Figure 1A) (Sridhar, 2018). Superficial cells harbor microvilli favoring exchanges with tear film and they also express membrane-associated mucins. The airway epithelium features ciliated cells, the most abundant cell type, secretory cells and basal cells, all attached to the basement membrane (Figure 1A). Cilia beat in a coordinated way within the PCL to move up the mucus layer (produced by secretory cells) toward the digestive system (Tarran, 2004). This phenomenon, called mucociliary clearance, is the first mechanism of defense against direct inhaled particles and pathogens embedded in mucus. Finally, the alveolar epithelium is comprised of a monolayer of two morphologically distinct types of cells, thin squamous alveolar type I (ATI) cells and cuboidal alveolar type II (ATII) cells.

Cutaneous, corneal and airway epithelia all include basal cells. Anchored to the basement membranes through hemidesmosomes and focal adhesions, these progenitor cells are characterized by high proliferative potential and the ability to migrate toward injured area (Barrandon and Green, 1987; Ladage et al., 2002; Fuchs and Nowak, 2008; Hackett et al., 2011; Mort et al., 2012). Basal cell populations of cutaneous, corneal and airway epithelia comprise stem/progenitor cells, which are precursors of keratinocytes, wing cells and ciliated/secretory cells, respectively. Although several different types of progenitor/stem cells (including basal, Club, neuroendocrine and ATII cells) have been identified in the lung, alveolar type II cells play a key role in alveoli, with the ability to give rise to new ATII or differentiate into ATI cells. Progenitor/stem cells are involved in the natural regeneration (self-renewal) and repair after damage of epithelial tissues. It has to be noted, however, that the cell turnover rates greatly differ among the different types of epithelia. While the corneal epithelium features rapid cell renewal (5–14 days) (Hanna and O'Brien, 1960; Sharma and Coles, 1989; Haddad, 2000), cutaneous cell turnover rates are much slower (36–56 days) (Halprin, 1972; Maeda, 2017). Although variable among studies and species, most estimates for cell turnover in adult airways and alveoli are higher than 100 days (Kauffman, 1980; Rawlins and Hogan, 2008; Rawlins et al., 2009). However, turnover times can markedly increase in response to injury.

Healthy epithelia have a remarkable ability to repair and regenerate after damage. The following series of cellular events are usually engaged sequentially: (1) cell spreading, (2) migration of healthy cells to the injured area, (3) cell proliferation and (4) differentiation. As detailed below (section Evidence for Epithelial Repair Impairment by *Pseudomonas aeruginosa*), *P. aeruginosa* infections impact these mechanisms, resulting in abnormal repair, and possibly remodeling of epithelia, ultimately altering their functions.

### *Pseudomonas aeruginosa* Bacteria

*Pseudomonas aeruginosa* is a motile gram-negative bacillus found almost everywhere in the environment such as in water, soil, plants and animals, including humans (Klockgether and Tümmler, 2017). *Pseudomonas aeruginosa* possesses a single polar flagellum responsible for swimming and swarming motility as well as for adherence to the epithelial cells (Köhler et al., 2000; Schwarzer et al., 2016). Several shorter type IV pili allow *P. aeruginosa* to attach to surfaces or epithelia and are responsible for their twitching motility (Kazmierczak et al., 2015).

*Pseudomonas aeruginosa* has an incredible ability to adapt to diverse and hostile environments. This property is possible because of its ability to form biofilms as well as genetic modifications/mutations and/or acquired antibiotic resistances due to antibiotic selection pressure that favors chromosomal mutations or horizontal transfer of resistance genes. This, in turn, results in a wide range of phenotypically different, and mostly rare, isolates. Hilker et al. compared the pathogenicity of 20 different environmental (from soil, plant and aquatic habitat) and clinical *P. aeruginosa* strains in an acute murine airway infection model (Hilker et al., 2015). Data highlighted a broad range of responses following infection, from no observed health consequences to 100% death rates. Moreover, results showed that strain pathogenicity did not segregate with the original environment, indicating that *P. aeruginosa* virulence is independent of its habitat.

*Pseudomonas aeruginosa* produces a multitude of pathogenicity factors during infections. Some are structural constituents and others are secreted or directly injected into host cells. Among structural constituents, *P. aeruginosa* flagellum and pili are responsible for motility and bacterial adhesion to host cells. In addition, lipopolysaccharide (LPS), a complex glycolipid, and lectins (LecA and LecB) are present in the outer membrane of *P. aeruginosa* and also contribute to its pathogenicity. Virulence factor production is coordinated by 3 major interrelated bacterial cell-to-cell communication systems, called quorum sensing: Las, Rhl, and PQS (Lee and Zhang, 2015). The types II and III (T2SS and T3SS) systems release the vast majority of known virulence factors. T2SS allows to secrete bacterial elastases, of which LasA and LasB elastases, proteases, enzymes, and pigments such as pyocyanin into the extracellular environment. T3SS is a needle-like complex of proteins used by *P. aeruginosa* to inject several bacterial cytotoxins directly into epithelial cells (Engel and Balachandran, 2009; Hauser, 2009; Galle et al., 2012). To date, four effectors of *P. aeruginosa* T3SS have been identified: exotoxin S (ExoS) and exotoxin T (ExoT) (bifunctional toxins with amino-terminal GTPase-activating proteins (GAP) activity and carboxy-terminal adenosine diphosphate ribosyl transferase (ADPRT) activity), exotoxin U (ExoU, phospholipase) and exotoxin Y (ExoY, adenylate cyclase). These numerous bacterial cell-associated constituents, secreted and injected virulence factors are involved in *P. aeruginosa* pathogenicity and can alter both epithelial integrity (see section Evidence for Epithelial Integrity Alterations by *Pseudomonas aeruginosa*) and repair efficiency (see section Evidence for Epithelial Repair Impairment by *Pseudomonas aeruginosa*).

*Pseudomonas aeruginosa* infections can be classified according to their type, i.e., acute vs. chronic (Furukawa et al., 2006; Valentini et al., 2018). Acute *P. aeruginosa* infections can spread rapidly, disseminate in the bloodstream, potentially leading to systemic infection and eventually to death within hours or days (McManus et al., 1985). Conversely, chronic *P. aeruginosa* infections develop more slowly and persist for months or years, as observed in cystic fibrosis patients (Heltshe et al., 2018). Isolated strains from acute and chronic infections feature different patterns of virulence (Burns et al., 2001; Smith et al., 2006; Feliziani et al., 2010). Indeed, acute infections usually require a functional T3SS, allowing *P. aeruginosa* to introduce toxins directly into host cells, and production of virulence factors such as quorum-sensing molecules, elastase, lipopolysaccharide, or type IV pili (Comolli et al., 1999; Turner et al., 2014). On the other hand, chronic infections generally involve biofilm formation and a global down regulation of genes coding for virulence factors and toxins (Martínez-Solano et al., 2008). However, T3SS and type VI secretion system (T6SS) effectors in *P. aeruginosa* isolates have been recovered both from acute pulmonary infections and chronic infections in CF patients (Boulant et al., 2018).

As opposed to the free-floating planktonic mode, biofilms are organized in surface-attached communities of *P. aeruginosa* bacteria, embedded in an extracellular matrix made of nucleic acids, polysaccharides, rhamnolipids, and siderophores (such as pyoverdine) (Mann and Wozniak, 2012; Maunders and Welch, 2017). Despite antibiotic therapy, *P. aeruginosa* within biofilms, which protect bacteria from the action of immune cells, remain difficult to eradicate, favoring the appearance of antibiotic resistance. Therefore, novel therapies, as adjuvant to antibiotics, are urgently needed to fight chronic *P. aeruginosa* infections, particularly within the biofilms.

### Cutaneous *Pseudomonas aeruginosa* Infections

Infections of the skin usually occur through damaged epithelial barriers, for example after traumatic, burn or surgical wounds as well as around implanted or indwelling devices (Weinstein and Mayhall, 2003; Turner et al., 2014) (Table 2). *Pseudomonas aeruginosa* is a natural member of the skin microflora in humans and is rarely responsible for infections in healthy people. However, this pathogen is one of the most commonly isolated bacteria in chronic wounds (Altoparlak et al., 2004; Gjødsbøl et al., 2006; Kirketerp-Møller et al., 2008). Elderly (>65 years) and immunocompromised patients as well as patients with an underlying pathology such as diabetes or vascular diseases, who are at risk of developing chronic ulcers of the skin, have higher risk of developing infection (Serra et al., 2015; Prevaldi et al., 2016).

**Table 2:** Risk factors for *P. aeruginosa* infections of the skin, the eye and lungs.

**Cutaneous infections**	**Corneal infections**	**Alveolar/airway infections**
Traumatic, burn, surgical wounds; Implanted or indwelling devices; age>65 years; immunodeficiency; diabetes; vascular diseases	Contact lens wear; ocular trauma, surgery, pre-existing corneal abnormalities;	Mechanical ventilation; immunodeficiency; chronic pulmonary diseases (COPD, CF)

An efficient healing process of an aseptic cutaneous wound involves first, coagulation, then inflammation, cell proliferation, and matrix repair to form a granulation tissue, and finally, re-epithelialization and remodeling or scar formation. As explained below (section Evidence for Epithelial Repair Impairment by *Pseudomonas Aeruginosa*), infections with *P. aeruginosa* bacteria can result in the failure of cutaneous injuries to heal, thus becoming chronic wounds. It has been shown that in these chronic wounds, *P. aeruginosa* usually attaches to the surface of the cutaneous epithelium and forms biofilms (Mihai et al., 2014; Roy et al., 2014; Brandenburg et al., 2015; Jensen et al., 2017). Bacteria are also able to invade the tissue more deeply (Figure 1B), where they have been observed in clusters, embedded in the extracellular matrix, or internalized in keratinocytes (Kirketerp-Møller et al., 2008; Zhao et al., 2010; Alves et al., 2018). Using a three-dimensional model of reconstituted human epidermis, Garcia et al. showed that both *P. aeruginosa* wt strain and flagellin mutant can adhere to the stratum corneum. However, flagellum plays a critical role in whole epidermis tissue invasion (including the stratum basale) as well as cytokine/chemokine induction, through the toll-like receptor 5 (TLR5), after keratinocyte infection (Garcia et al., 2018). Their *in vivo* data using a model of *P. aeruginosa* subcutaneous infection also suggested a key role of flagellum in the cutaneous persistence *of P. aeruginosa* (Garcia et al., 2018). Once established, it is almost impossible to eradicate *P. aeruginosa* with antibiotics in chronic wounds, which characteristically have a significantly greater area, and display delayed healing as well as persistent and uncontrolled inflammation (Gjødsbøl et al., 2006; Chaney et al., 2017).

### Ocular *Pseudomonas aeruginosa* Infections

*Pseudomonas aeruginosa* infections of the cornea are a major cause of blindness and visual impairment. Ocular trauma or surgery, pre-existing corneal abnormalities and contact lens wear are the major causes of keratitis. Moreover, *P. aeruginosa* is the most common pathogen isolated from cultures of corneal scrapings (Green et al., 2008) (Table 2). Without an efficient antibiotic treatment and an adequate control of the host inflammatory response, corneal tissue destruction can happen, favoring corneal ulcers, often leading to corneal scarring and reduced visual acuity, and potentially to enucleation (Constantinou et al., 2009).

From previous studies, using experimental infections in cellular or animal models or in patients wearing corneal lens, it has been established that *P. aeruginosa* is able to bind and invade corneal epithelial cells (Stern et al., 1985; Fleiszig et al., 1994, 1995; Ladage et al., 2002; Mun et al., 2013; Posch et al., 2014). Wounded corneal epithelia, and especially migrating cells, appear to be more susceptible to *P. aeruginosa* infection than intact epithelia (Spurr-Michaud et al., 1988; Fleiszig et al., 1997a). Fleiszig et al. demonstrated that exposed basolateral membranes of the polarized cells following injury are more susceptible to *P. aeruginosa* invasion than apical membranes (Fleiszig et al., 1997a). *Pseudomonas aeruginosa* can either penetrate the multilayered corneal epithelium or invade the corneal stroma through the injured corneal epithelium, contributing to the loss of corneal transparency and the development of corneal opacification (Cowell et al., 1998; Tam et al., 2010).

Different bacterial constituents may be involved in corneal epithelial invasion and colonization (Figure 1B). It has been shown for example that pili mutants of *P. aeruginosa* are associated with reduced invasion of corneal epithelial cells *in vitro* and decreased ability to colonize the cornea *in vivo* (Zolfaghar et al., 2003). Using a murine model of keratitis, it has been described that following *P. aeruginosa* stromal invasion, *P. aeruginosa* LPS and flagellin activate macrophages, resulting in the transcription of pro-inflammatory cytokines and chemokines, neutrophil infiltration and *P. aeruginosa* phagocytosis (Sun et al., 2010). The importance of epithelial cell response has also been emphasized through *in vitro* and *in vivo* models of keratitis. It has been shown for example that the corneal epithelial cell response to *P. aeruginosa* is regulated by the macrophage migration inhibitory factor (MIF), which promotes pro-inflammatory cytokine synthesis and bacterial invasion of corneal cells *in vitro* (Gadjeva et al., 2010). Indeed, reduced inflammatory response (neutrophil infiltration and cytokine levels) and improved bacterial clearance was observed after MIP inhibition *in vivo* (Gadjeva et al., 2010). Among the pro-inflammatory cytokines, interleukin 1β, which is upregulated by *P. aeruginosa* infection, is a key regulator of corneal immune response (Rudner et al., 2000). CCL2 and CCL3 chemokines also mediate neutrophil recruitment, as shown in a mouse model of *P. aeruginosa* keratitis (Xue et al., 2007). Then, neutrophil degranulation, release of proteolytic enzymes, such as matrix metalloproteinases (MMP), and production of reactive oxygen species can induce major tissue injuries (including epithelial abrasion, degeneration and necrosis), as shown in keratitis models (Banin et al., 2008; Aldebasi et al., 2014).

### Respiratory *Pseudomonas aeruginosa* Infections

Acute *P. aeruginosa* respiratory infections (Sawa, 2014) are commonly observed in mechanically ventilated patients (Parker et al., 2008) due to airway epithelial damage by endotracheal tubes. *Pseudomonas aeruginosa* ventilator-associated pneumonia cases are associated with mortality rates reaching up to 30% (Luyt et al., 2014). Patients with compromised lung defense are also prone to *P. aeruginosa* respiratory infections, especially neutropenic (Chatzinikolaou et al., 2000) and HIV (Sorvillo et al., 2001) patients.

When first acquired, *P. aeruginosa* respiratory infections are classified as “acute” (Sawa, 2014). However, eradication failure can lead to chronic *P. aeruginosa* infections and bacterial colonization. The leading causes allowing *P. aeruginosa* to colonize airways are either related to immunologic impairment/immunodeficiency or altered ability to clear pathogens (dysfunctional mucociliary clearance) due to airway epithelial injuries or remodeling in patients with chronic pulmonary diseases (Table 2). In this group of patients, intermittent episodes of increasing pulmonary symptoms, named “exacerbations,” occur and have been related to clonal expansion of *P. aeruginosa* strains colonizing patients (Aaron et al., 2004). Thus, chronic *P. aeruginosa* respiratory infections are frequently reported in patients with cystic fibrosis, chronic obstructive pulmonary disease or bronchiectasis (Martínez-Solano et al., 2008; Salvatore et al., 2011; Polverino et al., 2015). In the lungs of these patients, *P. aeruginosa* can persist for years, growing in biofilms and engaging multiple mechanisms to evade immune response of the host. *Pseudomonas aeruginosa* respiratory infections are also frequently observed in lung transplants (Bonvillain et al., 2007).

Several studies demonstrated that the cells at the wound edges of injured airway epithelia and the denuded basement membranes are more susceptible to binding by *P. aeruginosa* (Plotkowski et al., 1992; Tsang et al., 1994; Schwarzer et al., 2016). More specifically, it has been shown that *P. aeruginosa* adheres more frequently to mucus (Tsang et al., 1994) and extruded/dead cells (Tsang et al., 1994; Schwarzer et al., 2016), as well as basal and migrating flattened cells with lamellipodia (Plotkowski et al., 1992; de Bentzmann et al., 1996; Roger et al., 1999). Amino-acid sensor-driven chemotaxis and flagella-driven swimming appear to be the mechanisms by which *P. aeruginosa* reaches the cells at the wound edge of airway epithelia (Schwarzer et al., 2016). Finally, fibronectin, asialoGM1 and α5β1integrin may mediate *P. aeruginosa* binding to the airway epithelial cells Plotkowski et al., 1992; Saiman and Prince, 1993;Roger et al., 1999.

It has been shown that type IV pili-associated PilY1, pili retraction and T3SS are required for adherence of *P. aeruginosa* to injured human airway cell cultures and then bacterial invasion (Heiniger et al., 2010). Golovkine et al. proposed a chronological view of *P. aeruginosa* transmigration across kidney MDCK and alveolar A549 model epithelia (Golovkine et al., 2016). They showed that *P. aeruginosa* bacteria take advantage of cell division or sites of senescent cell extrusion, associated with cell-cell junctions alterations, to transmigrate across the epithelia. This is followed by a rapid and massive bacterial infiltration of the basolateral compartment. Bacteria then interact with their pili and inject T3SS toxins into host cells, inducing cytoskeletal disruption and cell detachment from the extracellular matrix, promoting rapid bacterial propagation under the cells (Figure 1B).

As shown in different animal models, acute *P. aeruginosa* pneumonia cause airway and alveolar epithelia injury, increased lung permeability and lung edema development (Wiener-Kronish et al., 1993; Kudoh et al., 1994; Finck-Barbançon et al., 1997; Pankhaniya et al., 2004; Rejman et al., 2009; Junkins et al., 2014; Sawa, 2014). Moreover, Cigana et al. showed that chronic infection with *P. aeruginosa* CF-adapted variants in mice leads to CF features of airway remodeling, including epithelial cell hyperplasia, goblet cell metaplasia and elevated levels of tissue damage markers (Cigana et al., 2016).

## Evidence for Epithelial Integrity Alterations by *Pseudomonas aeruginosa*

### Epithelial Cell Death, Cell Morphological Changes, and Altered Epithelial Integrity Induced by *Pseudomonas aeruginosa*

Epithelial cell death after exposure to either live *P. aeruginosa* bacteria or secreted virulence factors has been reported in numerous studies (Figure 1B). Indeed, a cytotoxic effect has been observed on human epidermal keratinocytes (Loryman and Mansbridge, 2008; Jeffery Marano et al., 2015; Chung et al., 2017) as well as corneal (Fleiszig et al., 1996; Ramirez et al., 2012) and airway/alveolar (Bajolet-Laudinat et al., 1994; Britigan et al., 1997; Gellatly and Hancock, 2013; Li et al., 2014) epithelial cells. Moreover, cell loss/detachment has been observed both in non-differentiated alveolar (Geiser et al., 2001) or differentiated airway epithelial cell cultures (Bajolet-Laudinat et al., 1994; Garcia-Medina et al., 2005). Interestingly, Tsang et al. showed an increased number of extruded cells from the epithelial surface in an adenoid nasal tissue and a complete disintegration of the epithelial structure, at 8 and 12 h, respectively, after infection with a clinical isolate of a non-mucoid, piliated strain of *P. aeruginosa* (Tsang et al., 1994).

Using three-dimensional tissue-engineered models of human skin wounds, it has been shown that *P. aeruginosa* colonized the upper epidermal layers before invasion into the dermis, caused a loss of epidermis and de-keratinization of the skin constructs, as well as partial loss of basement membrane (Shepherd et al., 2009). A decrease in transepithelial resistance, indicating a loss of epithelial integrity has also been observed in the human corneal (Ramirez et al., 2012) and mouse tracheal (Garcia-Medina et al., 2005) epithelia. In a mouse model of acute lung infection, Finck-Barbançon et al. also showed that *P. aeruginosa* disrupts the alveolar epithelium (Finck-Barbançon et al., 1997). Accordingly, several studies reported that *P. aeruginosa* infection is associated with the disruption of cell-cell contacts and loss of cellular junctions (Tsang et al., 1994; de Bentzmann et al., 2000; Geiser et al., 2001; Losa et al., 2015). The importance of epithelial polarity has been showed by the cytotoxic effect of the *P. aeruginosa* quorum-sensing molecule N-3-oxo-dodecanoyl-L-homoserine lactone (3-Oxo-C12-HSL) which altered airway epithelial integrity and gap junctions in non-polarized cells or wounded polarized cultures, in contrast with intact polarized epithelia (Losa et al., 2015). Moreover, impaired epithelial integrity is accompanied with increased cytotoxic effect of *P. aeruginosa* on host epithelial cells (Fleiszig et al., 1997a; Lee et al., 1999) (Figure 1B). In some cases, aggressive, recalcitrant *P. aeruginosa* sinonasal infections have been shown associated with severe tissue necrosis (Kuan et al., 2017).

**Figure 2:**
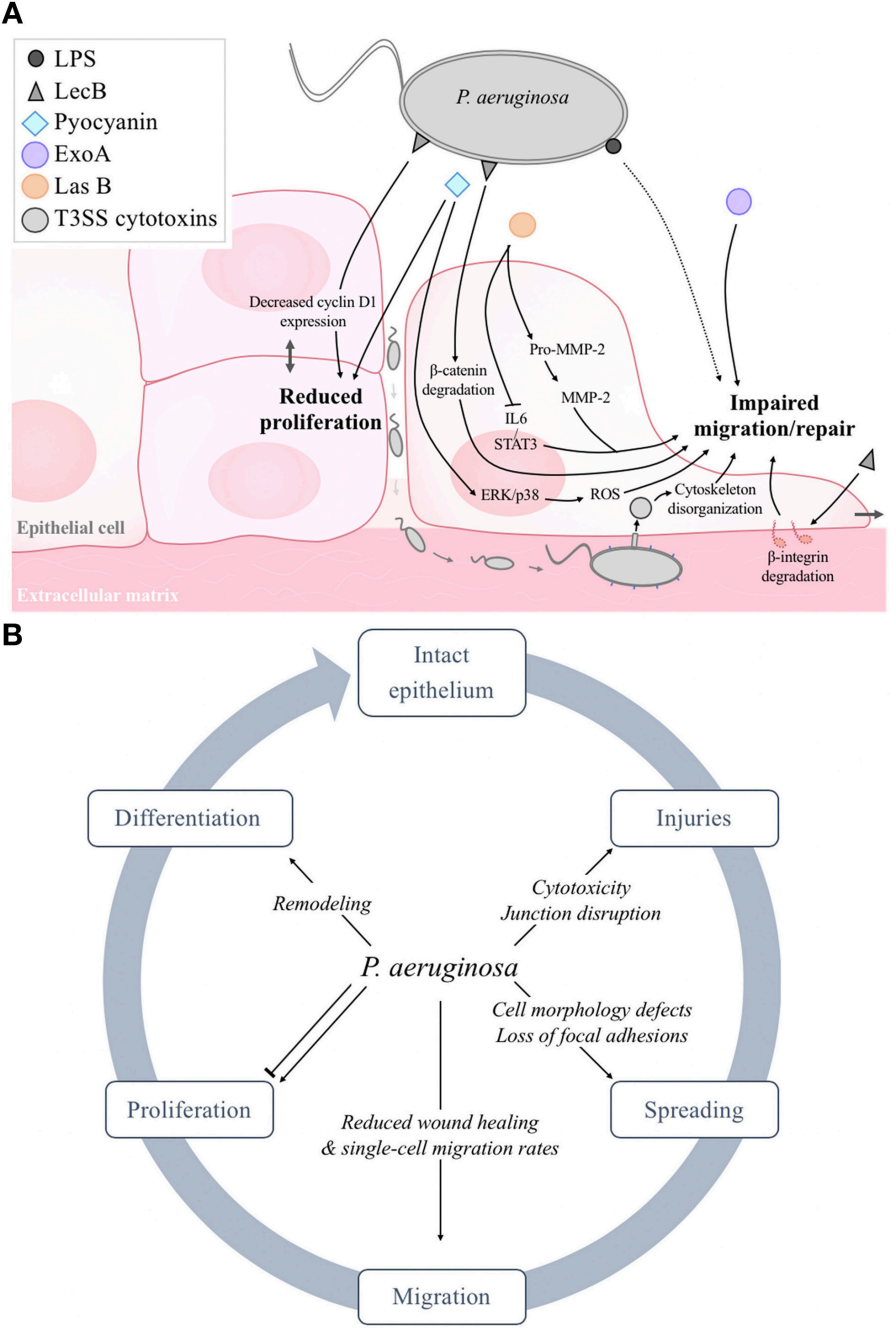
**(A)** Schematic model of the negative effects of *P. aeruginosa* structural constituents (LPS, LecB) and exoproducts (pyocyanin, ExoA, LasB, LasA, T3SS exotoxins) on epithelial cell proliferation, migration and wound repair. As reported in section Evidence for Epithelial Repair Impairment by *Pseudomonas aeruginosa*, it has to be noted that some factors (LPS, flagellum, 3-oxo-C_12_-HSL) can also elicit stimulatory effects. **(B)** Summary of the deleterious effects of *P*. *aeruginosa* on epithelial integrity and repair.

Finally, it has been shown that *P. aeruginosa* alters cell morphology in cutaneous, corneal and airway epithelia (Geiser et al., 2001; Jeffery Marano et al., 2015; Losa et al., 2015) (Figure 2). This aspect has been more extensively studied in the airway epithelium. Indeed, several groups described that epithelial cells at the wound edges of injured airway epithelia exposed to *P. aeruginosa* bacteria or *P. aeruginosa* virulence factors feature reduced or loss of lamellipodial structures, stress fibers, focal adhesions and destruction of the actin cytoskeleton (de Bentzmann et al., 2000; Geiser et al., 2001; Losa et al., 2015) (Figure 1B). In a novel murine model of contact lens wear, it has been shown that PAO1 inoculation is associated with altered cell morphology, disrupted basal membrane and disorganized epithelial structure, with or without corneal opacity (Metruccio et al., 2019).

### Bacterial Factors Responsible for Alteration of Epithelial Integrity

*Pseudomonas aeruginosa* structural constituents are detrimental for epithelial integrity (Figure 1B). Accordingly, LPS from *P. aeruginosa* is frequently used in *in vivo* studies of induced acute lung injury. *In vitro*, it has been shown for example that LPS-exposed A549 (alveolar) and BEAS-2B (bronchial) cells display morphological changes, cell apoptosis and reduced cell survival (Cabrera-Benítez et al., 2016); these effects were attenuated by pyrrole compounds, which also dampened the inflammatory response. In addition, Yi et al. showed that LPS induced a decrease in corneal transepithelial resistance, through tight junction disruption, by targeting ZO-1 and ZO-2 proteins (Yi et al., 2000). It has also been reported that LecA- and LecB- producing *P. aeruginosa* strains induce cytotoxic effects on A549 cells and are associated with higher alveolar barrier impairment than *lecA* or *lecB* mutants in a model of acute lung injury (Chemani et al., 2009). Moreover, co-treatments with lectin inhibitors reduced lung injury and mortality. The role of proteins involved in pili formation and function in mediating epithelial injury has also been studied. It has been reported (Comolli et al., 1999) that *pilT* or *pilU* mutants caused less cytotoxicity on A549 cells than the wild-type strain, but more than an isogenic, non-piliated *pilA* mutant.

Exotoxin A (ExoA) is a highly toxic virulence factor released by *P. aeruginosa* into the extracellular medium via the T2SS system (Michalska and Wolf, 2015). It has been shown that ExoA contributes to corneal (Twining et al., 1993) and airway (Plotkowski et al., 2002; Gellatly and Hancock, 2013) epithelial injuries by inducing cell death. Moreover, ExoA may contribute to the degradation of cornea during keratitis induced by *P. aeruginosa*, by inhibiting corneal protein synthesis and activating corneal proteases such as MMP-2 (Twining et al., 1993). Azghani et al. also showed that ExoA decreased transepithelial resistance and enhanced paracellular permeability of type II pneumocyte cultures on permeant filters, indicating altered epithelial integrity (Azghani, 1996). Similarly, rhamnolipids contribute to epithelial barrier disruption through tight-junction alterations (Zulianello et al., 2006). In addition, it has been established that the effect of the quorum sensing molecule 3-Oxo-C12-HSL on airway epithelial integrity is dependent on intracellular Ca^2+^ and is prevented by Src tyrosine- and Rho-associated protein kinase inhibitors (Losa et al., 2015). Another secreted factor, the LasB elastase, plays a major deleterious effect on epithelial integrity. Indeed, it alters the extracellular matrix of cutaneous, airway and corneal epithelia through degradation of type I and type IV collagen proteins (Bejarano et al., 1989; Nagano et al., 2001). Moreover, it has been shown that *P. aeruginosa* elastase promotes collagen degradation by inducing the conversion of the inactive precursors of several MMP to active enzymes (de Bentzmann et al., 2000; Nagano et al., 2001). In human airway epithelial cells, loss of stress fibers and depolymerization of the actin cytoskeleton after exposure to *P. aeruginosa* purified elastase was also observed (de Bentzmann et al., 2000). Finally, chronic lung exposure to pyocyanin in mice is associated with extended tissue damage and remodeling, characterized by goblet cell hyperplasia, fibrosis, and alveolar destruction (Caldwell et al., 2009).

Exotoxins from the T3SS system, once in the cytoplasm of host epithelial cells, induce cell death by necrosis or apoptosis (Finck-Barbançon et al., 1997; Pederson and Barbieri, 1998; Lee et al., 2005; Shafikhani et al., 2008; Hauser, 2009), thus favoring disruption of epithelial barriers (Shaver and Hauser, 2004). It has been shown that *P. aeruginosa* strains, producing ExoS but not ExoU (Fleiszig et al., 1997b), can invade and survive within epithelial cells (Fleiszig et al., 1994, 1996). This probably favors *P. aeruginosa* persistence and leads to host cell death. Such persistent survival of *P. aeruginosa* also likely alters epithelial integrity and repair processes, by interacting with intracellular proteins and/or structures. However, further studies are needed to confirm this hypothesis.

Although the T3SS pathogenicity has mainly been attributed to the ExoU effector, Ader et al. (2005) showed that *P. aeruginosa* strains producing ExoS and ExoT also induced cytotoxic effects *in vitro* on A549 cells and alveolar damage in a murine model of acute lung injury. It has also been shown that ExoS and ExoT elicits actin disruption and cytotoxic effects on eukaryotic cells through their N-terminal Rho GAP (targeting Rho, Rac and Cdc42 signaling pathways) and C-terminal ADPRT activities, while the adenylate cyclase activity of ExoY is associated with actin cytoskeleton disruption *in vitro* (Pederson et al., 1999; Garrity-Ryan et al., 2004; Cowell et al., 2005). Altogether, these studies clearly demonstrated that T3SS has a major role in epithelial damage. However, the relative contribution of T3SS exotoxins to damage severity as well as the precise molecular and cellular mechanisms associated with the observed epithelial alterations would deserve further study, using primary cutaneous, corneal and airway epithelial cultures.

## Evidence for Epithelial Repair Impairment by *pseudomonas aeruginosa*

### Impact of *Pseudomonas aeruginosa* on Cell Proliferation

Data from airway epithelial models, including studies from our group, showed that *P. aeruginosa* (either purified exoproducts or *P. aeruginosa* supernatants) elicits an inhibitory effect on airway epithelial cell proliferation (Cott et al., 2016; Ruffin et al., 2016).

In cutaneous models, *P. aeruginosa* supernatants significantly decreased the proliferation of human epithelial keratinocytes (Jacobsen et al., 2012; Jeffery Marano et al., 2015). However, *P. aeruginosa* lipopolysaccharide increased the proliferation of normal and transformed human keratinocytes, likely through NF-κB and cyclin D1 induction (Preciado et al., 2005). Moreover, *P. aeruginosa* strain PAO1 was found to promote epidermal cell proliferation and wound healing rates in rat cutaneous wounds, through the induction of inflammatory TNF-α responses (Kanno et al., 2013).

Therefore, although data from the literature point toward an inhibitory effect of *P. aeruginosa* on airway epithelial cell proliferation (Figure 2), a consensus has not been reached about the effect of *P. aeruginosa* on cell proliferation in cutaneous models. These contradictory results may be due to the different experimental conditions and models.

### *Pseudomonas aeruginosa* Impairs Migration and Wound Repair

Different *in vitro* epithelial cell models from simple cell line monolayers to fully differentiated primary cultures of epithelial cells and *in vivo* animal models have been used to study the impact of infection on cell migration and wound repair. Moreover, several “infection” protocols are commonly used to determine the impact of *P. aeruginosa* on epithelial repair. These protocols employed exposure of epithelial cells or epithelia to either live bacteria, filtrates of bacterial cultures containing a pool of *P. aeruginosa* secreted virulence factors (also called supernatants, filtrates, or conditioned media) or purified bacterial components or virulence factors. Mixture of secreted virulence factors (in bacterial filtrates) or purified virulence factors have been commonly used in the *in vitro* experiments (de Bentzmann et al., 2000; Brothers et al., 2015; Ruffin et al., 2016; Saint-Criq et al., 2017; Adam et al., 2018), probably because of the highly cytotoxic nature of the live bacteria on isolated epithelial cell cultures. Thus, some of the discrepancies in the results obtained by different groups can be explained, at least in part, by the differences in models and experimental protocols used.

Time-lapse videomicroscopy is a powerful approach to assess single-cell migration dynamics (cell spreading, motility, social behavior, etc.) as well as to observe a live-repairing epithelium. To the best of our knowledge, single-cell migration assays have been performed mostly using airway epithelial cells. We, and others, demonstrated that secreted virulence factors from laboratory or clinical *P. aeruginosa* strains altered cell migration (de Bentzmann et al., 2000; Ruffin et al., 2016). More specifically, we showed that *P. aeruginosa* exoproducts decreased the migration rates and increased the tortuosity of primary human airway epithelial cells, thus impairing the critical process of directional migration during repair (Ruffin et al., 2016).

The most frequently used *in vitro* method to assess cell migration and wound repair is to cause injuries on epithelial cell monolayers or fully differentiated epithelia, using mechanical (conventional scratch assay), chemical (1M NaOH), and electrical approaches. Measurements of “free of cells” areas, after injury and over the time during repair, thus allow to compare the wound repair rates, in various conditions (Ruffin et al., 2016). By using scratch assays on monolayer cultures of human keratinocytes (Loryman and Mansbridge, 2008; Jacobsen et al., 2012), corneal epithelial cells (Brothers et al., 2015) or alveolar/airway epithelial cells (Geiser et al., 2001; Cott et al., 2016; Ruffin et al., 2016; Saint-Criq et al., 2017; Adam et al., 2018), it is generally described that *P. aeruginosa* impairs the ability of epithelia to repair (Figure 2). As detailed below (section Molecular Mechanisms Responsible for Repair Impairment by *Pseudomonas aeruginosa*), various factors, including LPS (Loryman and Mansbridge, 2008), lectin B (Cott et al., 2016), and LasB elastase (Ruffin et al., 2016), have been shown to elicit an inhibitory effect on the wound repair of keratinocytes, lung epithelial cell lines, and primary human airway epithelial cell monolayers, respectively. Finally, we observed that the repair rates of the injuries performed on fully-differentiated airway epithelia grown on permeant filters at the air-liquid interface is significantly reduced after exposure to *P. aeruginosa* exoproducts (Ruffin et al., 2016).

Wound repair can also be assessed using more physiological models by performing wounds (of determined diameters), *ex vivo*, on explanted epithelial tissues, as well as *in vivo*, on live animals. It has been shown that *P. aeruginosa* laboratory or clinical strains decrease the repair rate of cutaneous wounds in rabbit, murine or porcine in *in vivo* models (Zhao et al., 2010; Mendes et al., 2013; Pastar et al., 2013; Brandenburg et al., 2015; Karna et al., 2016; Chaney et al., 2017). Conversely, Gao et al. observed that purified *P. aeruginosa* flagellin significantly accelerated the wound closure of porcine eye wounds (Gao et al., 2010). Furthermore, Chaney et al. observed that the reepithelialized part of the skin covering *P. aeruginosa* infected porcine wounds, were thicker and hyper-proliferative, compared to that in non-infected burn wounds (Chaney et al., 2017).

### Molecular Mechanisms Responsible for Repair Impairment by *Pseudomonas aeruginosa*

As described above (section *Pseudomonas aeruginosa* Infections in Cutaneous, Corneal, Airway, and Alveolar Epithelia), the important role of *P. aeruginosa* flagellum in bacterial motility and adhesion to wounded tissues has been established, but the impact of flagellum or flagellin on epithelial repair has not been extensively studied. Nevertheless, Gao et al. suggested that flagellin promotes epithelial repair in cultured human corneal epithelial cells and porcine corneas, through EGFR/TGFα-mediated signaling pathways (Gao et al., 2010).

Conflicting results on the impact of *P. aeruginosa* LPS on epithelial repair rates have been reported. Thus, LPS was shown to accelerate the wound repair of airway epithelial monolayers in one study (Koff et al., 2006), whereas another report indicated inhibitory effect of LPS on the wound repair of keratinocyte monolayers (Loryman and Mansbridge, 2008). Moreover, LPS-independent inhibitory effects of *P. aeruginosa* on epithelial repair are also possible, as indicated by a study from de Bentzmann et al. showing similar decrease in human airway epithelial cell migration velocity induced by supernatants from either wildtype or LPS-deficient *P. aeruginosa* strains (de Bentzmann et al., 2000). These apparent discordant observations on the role of LPS may be attributable to the differences between the models, measured outcomes and/or the used concentration of LPS.

Another bacterial cell constituent, LecB, has been shown to impair the repair processes (Figure 2A). Indeed, Cott et al. showed that LecB decreased the migration and proliferation of human lung epithelial cells and also cell-cell contacts (Cott et al., 2016). These behavioral changes were associated with increased NF-κB transcriptional activity, degradation of β1-integrin and β-catenin (through GSK-3β-activity) and reduced c-myc and cyclin D1 expression. Interestingly, they also showed that L-fucose, a synthetic glycoconjugate that binds specifically to LecB, prevents these LecB-induced changes in host cell behavior and signaling.

Various secreted virulence factors are also involved in the inability of infected wounds to repair (Figure 2A). Using a rat model of acute granulating cutaneous injury, Heggers et al., demonstrated that wound contraction was delayed in animals exposed to viable *P. aeruginosa* bacteria or only ExoA. This deleterious effect on wound closure was prevented by the inclusion of anti-ExoA (Heggers et al., 1992). With *in vitro* models of human alveolar cells (Muller, 2006) and cutaneous fibroblasts (Muller et al., 2009), Muller *et al*. showed that exposure to pyocyanin resulted in cell growth arrest, apoptosis and senescence as well as wound repair inhibition, probably through the induction of reactive oxygen species (Muller, 2006) and ERK/p38(MAPK) signaling (Muller et al., 2009).

To determine whether exoproducts under the control of quorum sensing systems are involved in cutaneous wound healing impairment, Jacobsen et al. compared the impact of exoproducts secreted by a wild-type *P. aeruginosa* strain and its isogenic mutant, deficient for *lasR* and *rhlR* genes (Jacobsen et al., 2012). They found that quorum-sensing inactivation prevents the inhibitory effect of *P. aeruginosa* exoproducts on keratinocyte cell migration and proliferation. Exoproducts under LasR control, such as LasA protease and LasB elastase, play a key role in the pathogenesis of *P. aeruginosa* (Lee and Zhang, 2015). To define if LasB elastase impacts airway epithelial repair, de Bentzmann et al. exposed human nasal epithelial cells to virulence factors secreted by *P. aeruginosa* elastase-deficient strains. They showed that virulence factors from these strains have no inhibitory effect on migration velocity of nasal epithelial cells during repair, unlike the wild-type *P. aeruginosa* strains, indicating a role for *P. aeruginosa* elastase in airway epithelial repair impairment (de Bentzmann et al., 2000). Finally, this study indicated that the altered airway wound closure induced by *P. aeruginosa* virulence factors could be due to an imbalance between pro- and activated forms of MMP-2.

We recently studied which exoproducts are responsible for airway epithelial repair impairment, either in non-differentiated or fully differentiated primary airway epithelial cell cultures from non-CF and CF patients (Ruffin et al., 2016). To achieve our goals, we used either *P. aeruginosa* purified elastase, or exoproducts from different *P. aeruginosa* strains, including laboratory wild-type and engineered mutants deficient for LasR or various virulence factors. Altogether, our results point toward a critical role of LasB elastase, and secondarily to LasA protease, under LasR control in the observed repair impairment of non-CF and CF airway epithelial repair. The contribution of elastase has been confirmed by Saint-Criq et al., who further described the mechanism involved, i.e., IL-6/STAT3 signaling pathway down-regulation by LasB elastase (Saint-Criq et al., 2017). Our work (Ruffin et al., 2016) also indicated that *P. aeruginosa* strains may have variable impact on airway wound repair as a function of their genotypic, and consequently phenotypic characteristics. Indeed, exoproducts from a natural Early isolate (during early intermittent infection) from a 6-month-old CF patient severely impaired airway epithelial repair, whereas exoproducts from a clonally related Late isolate (chronic infection with a CF-adapted strain, harboring 68 mutations including in the *lasR* gene) from the same patient at 8 years of age, did not impact the repair rates.

Contrary to the vast majority of virulence factors altering epithelial repair, several lines of evidence indicate that the Las quorum sensing system molecule 3-Oxo-C_12_-HSL improves cutaneous wound healing and reepithelialization (Nakagami et al., 2011; Paes et al., 2012; Kanno et al., 2013). Nakagami et al. suggested that the presence of 3-Oxo-C_12_-HSL accelerates rat cutaneous wound healing by favoring fibroblast differentiation (Nakagami et al., 2011).

The role of T3SS cytotoxins has also been studied (Figure 2A). Using a model of diabetic mice, Goldufsky et al. showed that back skin wounds infected with the wild-type *P. aeruginosa* strain 103 (PA103), harboring functional T3SS, failed to heal, unlike the non-infected wounds or wounds infected with an isogenic T3SS-defective PA103 mutant (Goldufsky et al., 2015). More specifically, Geiser et al. demonstrated repair impairment of A549 alveolar cell monolayers in the presence of ExoT, secondary to actin cytoskeleton disorganization, loss of stress fibers and lamellipodial structures, cell rounding and detachment (Geiser et al., 2001). However, it should be noted that most of the studies on the impact of cytotoxins on host epithelia have been performed using immortalized epithelial cell lines. Further experiments using differentiated cultures of primary human epithelial cells from the skin, eyes or airways as well as *in vivo* models will be essential to understand the effects of T3SS and its effects on epithelial repair processes.

## Strategies to Counteract the Deleterious Impact of *Pseudomonas aeruginosa* on Epithelial Integrity and Repair

The most obvious way to avoid the damaging impact of *P. aeruginosa* on epithelia is to kill the bacteria. Antibiotics thus remain the first line of treatment in *P. aeruginosa* infections. However, with the recrudescence of multi-drug resistant strains, bacteria are more difficult to eradicate, and chronic infections may develop. Moreover, because exposure to *P. aeruginosa* virulence factors can rapidly damage epithelia, the development of complementary approaches, which can be used as antibiotic adjuvant, are required to limit injuries and favor epithelial repair. Here, we focus on these complementary approaches, while new antibiotic therapies, already discussed in numerous reviews (Langton Hewer and Smyth, 2017; Bassetti et al., 2018) will not be presented.

### Bacteriophages

Bacteriophages are viruses that specifically infect bacteria, by binding to their surface receptors. After penetration, they can induce bacterial death, irrespective of antibiotic resistances. Some evidence, described below, suggests that bacteriophage therapy may be an interesting strategy in *P. aeruginosa* infected wounds in cutaneous, ocular, and respiratory epithelial tissues.

In rodent and pig models, Mendes and colleagues showed that topical bacteriophage treatment significantly decreased bacterial colony counts and improved the healing of diabetic cutaneous wounds (Mendes et al., 2013). Another study showed that wound dressing in combination with honey, polyvinyl alcohol, chitosan nanofibers loaded with bee venom and bacteriophage against *P. aeruginosa* exhibited the most potent antibacterial activity against *P. aeruginosa* and improved wound healing compared to other formulations of wound dressing without bacteriophages (Sarhan and Azzazy, 2017). In a mouse model of *P. aeruginosa* keratitis, it has been shown that administration of eye-drops containing bacteriophages efficiently eradicated bacteria and allowed to preserve the structural integrity and transparency of the cornea, including the corneal epithelium (Fukuda et al., 2012). Although bacteriophage therapy for *P. aeruginosa* respiratory infections have demonstrated an antibacterial effect (Alemayehu et al., 2012; Kalia, 2013; Waters et al., 2017) and reduction in lung damage (Chang et al., 2017), so far there are, to the best of our knowledge, no studies specifically assessing the beneficial effect of bacteriophages during airway epithelial repair.

### Quorum Sensing Inhibitors

As previously described, the quorum sensing autoinducer 3-oxo-C_12_-HSL has been shown, on one hand, to contribute to the cytotoxic effect of *P. aeruginosa* on airway epithelial cells (Losa et al., 2015) but, on the other hand, to promote the healing of cutaneous wounds (Nakagami et al., 2011; Paes et al., 2012; Kanno et al., 2013). Thus, this compound may be considered for the treatment of chronic, non-healing and infected cutaneous wounds. However, further studies are needed to assess its cytotoxic vs. pro-repair effects on epithelial tissues.

It has been suggested that use of quorum sensing inhibitors (molecules inhibiting virulence factors production and biofilm formation without affecting bacterial viability), as adjuvant to antibiotic therapy, may be an interesting strategy for the treatment of bacterial infections (Bhardwaj et al., 2013; Kalia, 2013). However, their ability to counteract the negative effects of infection on epithelial repair has not been extensively assessed. We recently provided the first proof-of concept evidence that a quorum sensing inhibitor, called HDMF (4-hydroxy-2,5-dimethyl-3(2H)-furanone), prevents the deleterious impact of *P. aeruginosa* secreted virulence factors on airway epithelial repair (Ruffin et al., 2016). Indeed, we demonstrated that human airway epithelial cell monolayers and fully differentiated primary human bronchial epithelial cell cultures from non-CF and CF patients exposed to supernatants from bacterial cultures grown in the presence of HDMF featured similar repair rates as measured in the absence of bacterial products, whereas the repair rates of cell cultures exposed to virulence factors from *P. aeruginosa* cultures without HDMF were severely impaired.

### Anti-microbial Peptides

Antimicrobial peptides are naturally present in living organisms and are produced, and released, by neutrophils and epithelial cells in human skin, lungs and cornea in response to infection but can also be synthetically produced. These anti-microbial peptides are promising therapeutic options to treat *P. aeruginosa* infections as they allow to lower the negative impact of virulence factors on host cells and favor skin epithelial repair (Pfalzgraff et al., 2018) with low resistance rates.

Numerous antimicrobial peptides have been discovered or synthesized. Among them, the human cathelicidin LL-37 antimicrobial peptide has been extensively studied. It has been shown, that this peptide elicits both antimicrobial and antibiofilm activities (Dean et al., 2011) and enhances airway cell proliferation (Shaykhiev et al., 2005) and epidermal and ocular cell migration (Tokumaru et al., 2005; Huang et al., 2006), thus promoting wound healing. Moreover, Chung et al. showed that a Komodo dragon derived synthetic peptide called DRGN-1 improved the healing of murine cutaneous wounds infected with a mixed biofilm of *P. aeruginosa* and *Staphylococcus aureus* with a better efficiency than LL-37 (Chung et al., 2017).

Synthetic anti-lipopolysaccharide peptides (SALP), such as Pep19-2.5 or Pep19-4LF, have been shown to increase keratinocyte migration without affecting proliferation (Pfalzgraff et al., 2016). CAP37, which possess a LPS-binding activity, is bactericidal and accelerates the healing of infected corneal wounds *in vivo* (Kasus-Jacobi et al., 2015).

While cutaneous and corneal wounds can be easily treated with antimicrobial peptides by using topical solutions or wound dressings, direct lung delivery of antimicrobial peptides to treat respiratory infections is more challenging. Additionally, access to the epithelium is limited by the mucus layer covering the airways. Nevertheless, Chen et al. recently reported a beneficial effect of a frog skin-derived antimicrobial peptide, named Esc (1-21), in an *in vivo* murine model of pulmonary infection by *P. aeruginosa* (Chen et al., 2017). Moreover, transepithelial resistance of airway epithelial cell cultures was more efficiently preserved by this peptide than with LL-37. Inhalable formulations of antimicrobial peptides, as developed, for example by d'Angelo et al. (2015), may thus be of interest to counteract the deleterious impact of *P. aeruginosa* during respiratory infections.

### Photodynamic Therapy

Photodynamic therapy requires the use of photosensitizers, injected intravenously or applied to an infected site, and subsequently incorporated by bacteria. After activation of these photosensitizers with light of specific wavelength, the secondary production of reactive oxygen species (ROS) is associated with bacterial death (Hamblin and Hasan, 2004). Katayama et al. showed that photodynamic therapy significantly accelerated the wound healing of *P. aeruginosa*-infected cutaneous ulcers in mice, probably through the bactericidal impact of this treatment and its effect on biofilm production (Katayama et al., 2018). The authors also showed that photodynamic therapy was more efficient than classical antibiotic treatment with piperacillin-tazobactam, which failed to decrease bacteria counts in ulcer tissue and to improve healing. Recently, Grzegorz et al. demonstrated that antimicrobial blue light has the potential to inhibit the formation of biofilms from different *P. aeruginosa* clinical and laboratory strains *in vitro* and to reduce the virulence in a *Caenorhabditis elegans in vivo* infection model (Grzegorz et al., 2018). Thus, photodynamic therapy could be an interesting approach to favor epithelial repair in patients with multi-resistant *P. aeruginosa* cutaneous infected wounds. Interestingly, this strategy has also been considered in the treatment of airway infections in cystic fibrosis patients (Cassidy et al., 2011) using longer wavelengths to allow deeper tissue penetration.

### Azithromycin

Slater et al. showed that azithromycin increased epithelial resistance and decreased epithelial permeability of primary bronchial epithelial cell cultures on permeant filters at air-liquid interface at different stages of differentiation (Slater et al., 2016). These data suggest that azithromycin may favor reepithelialization of an injured airway epithelial barrier. They also demonstrated that azithromycin pre-treatment limits the decrease in transepithelial electrical resistance following LPS exposure.

### Anti-lectins

As described above, lectins, which are cell-associated constituents of *P. aeruginosa*, contribute to altered epithelial repair processes. In an *in vivo* murine model of acute lung injury, Chemani et al. showed that specific inhibition of lectins A and B with carbohydrates reduced lung injury and mortality in mice (Chemani et al., 2009). A case report also described a successful treatment of *P. aeruginosa* airway infection, not resolved by antibiotic treatments, with inhalations of galactose and fucose, which are specific lectins sugars (von Bismarck et al., 2001). This was, to the best of our knowledge, the first report of an effective lectin inhibition-based therapy. More studies are required to determine whether this positive impact was due to an antibacterial effect alone or to any other effects restoring airway epithelial integrity and function.

### Lipoxin A_4_

Several studies showed that lipoxin A_4_ (LXA_4_), a lipid mediator, may improve the healing of sterile cutaneous (Reis et al., 2017) and corneal (Kakazu et al., 2012) wounds. Moreover, Higgins et al. have showed that LXA_4_ protects the airway epithelium from tight junction disruption induced by *P. aeruginosa* infection and ultimately delays *P. aeruginosa* invasion (Higgins et al., 2016). Altogether, these results suggest that LXA_4_ could be a potential therapeutic option to treat *P. aeruginosa* cutaneous, corneal and airway infected wounds.

### CFTR Activation

Cystic fibrosis transmembrane conductance regulator (CFTR) encodes a chloride channel, expressed in various epithelia, which is defective in patients with cystic fibrosis. Our laboratory and others previously highlighted that CFTR function is crucial for cutaneous, corneal and airway epithelial repair processes under non-infectious conditions (Cao et al., 2010; Schiller et al., 2010; Trinh et al., 2012; Dong et al., 2015). We also showed that the repair of CF airway epithelia is defective, compared to a healthy epithelium. However, our work revealed for the first time that the functional rescue of CFTR in CF airway epithelial cells, with CFTR correctors (Vx-325 or Vx-809) and potentiator (Vx-770) improved the repair rates (Trinh et al., 2012; Adam et al., 2018). Although the efficiency of these molecules to rescue CFTR is dampened by *P. aeruginosa* (Stanton et al., 2015; Trinh et al., 2015; Maillé et al., 2017), a slight but significant improvement in airway wound repair rates was observed in the presence of CFTR modulators, despite the presence of *P. aeruginosa* exoproducts (Adam et al., 2018). It has to be noted, however, that increased CFTR expression in corneal cells in hypoxic conditions is associated with exacerbated binding and internalization of *P. aeruginosa* (Zaidi et al., 2004). Moreover, disruption of CFTR-dependent lipid rafts reduced *P. aeruginosa* internalization and cytotoxic effects (Zaidi et al., 2008). Therefore, further studies are needed to clearly establish the complex role of CFTR in the integrity and repair of various epithelia in the presence of *P. aeruginosa* infection.

## Current Limitations and Future Developments

Several mechanisms involved in epithelial repair, including cytoskeleton organization, cell migration and proliferation, have been shown to be affected by *P. aeruginosa* infection (Figure 2B). While differentiation is described as an important step for the restoration of epithelial integrity and function after injury, only few studies specifically addressed the impact of *P. aeruginosa* on differentiation of cutaneous, corneal or airway epithelia. This can be partly explained by the experimental time needed to study this last step of wound repair and the complexity of required models. Indeed, most of the studies on epithelial repair were performed on non-differentiated monolayers of cell lines and primary cultures. However, the contribution of several cell types (see section *Pseudomonas aeruginosa* Infections in Cutaneous, Corneal, Airway, and Alveolar Epithelia) in the multifaceted process of epithelial repair leading to the restoration of a differentiated epithelium further requires the use of complex *in vivo* models. While such models can be quite easily used for skin and ocular infections studies; the impact of *P. aeruginosa* on the alveolar and airway epithelia *in vivo* is particularly difficult to evaluate due to its limited access. The use of fully differentiated primary airway epithelial cultures at the air-liquid interface thus offers an alternative to study the impact of respiratory infections on airway repair (Losa et al., 2015; Higgins et al., 2016; Ruffin et al., 2016; Slater et al., 2016; Adam et al., 2018).

A huge number of bacterial constituents and secreted virulence factors have been shown to be involved in the deleterious impact of *P. aeruginosa* on either cutaneous, corneal, alveolar, or airway epithelia. However, it remains to be defined if these compounds share the same mechanisms of action, and downstream signaling pathways, within all these epithelia. Therefore, additional studies are needed to answer to these important questions. Moreover, because some specific features characterize skin, cornea, alveolar, and airway epithelial repair processes, therapeutic molecules or approaches aimed at counteracting the effect of bacteria and then improving repair processes should be validated in each of these different tissues. Experimental approaches should also take into account that it is important to define whether the beneficial impacts of treatments are mainly due to a reduction in bacterial counts, thus decreasing the bacterial-induced damage, or to a change in bacterial behavior, thus counteracting its deleterious effect on epithelial repair processes. Finally, as *P. aeruginosa* is often associated with other bacteria in chronic infected wounds (Choi et al., 2018), it would be important to further study the impact of infection on epithelial repair using more complex models of co-infections.

## Conclusion

Altogether, cutaneous, ocular, and respiratory *P. aeruginosa* infections are responsible for a large number of hospitalizations, disabilities, and deaths worldwide. Since the last few decades, an alarming recrudescence of resistant and multi-resistant isolates of *P. aeruginosa* has been observed. Moreover, it has been clearly established that *P. aeruginosa* infections cause both epithelial injuries and epithelial repair impairment (Figures 1, 2), making it easier, for bacteria, to colonize tissues, and more difficult, for the host, to eradicate bacteria and maintain organ functions. Thus, adjuvant therapies to antibiotics favoring epithelial integrity and repair despite the presence of infection, appear to be an important and promising therapeutic development avenue. Such strategies are critical to avoid this viscous circle of infection, causing injuries, then decreasing the epithelial function of defense against pathogens, ultimately leading to chronic infections and non-healing wounds.

## Author Contributions

All authors listed have made a substantial, direct and intellectual contribution to the work, and approved it for publication.

### Conflict of Interest Statement

The authors declare that the research was conducted in the absence of any commercial or financial relationships that could be construed as a potential conflict of interest.
